# A comic that explains palliative care: how patients experience comic-based educational material

**DOI:** 10.1007/s00508-024-02480-9

**Published:** 2025-01-07

**Authors:** Anna Kitta, Sarah Winsauer, Sal Marx, Lea Kum, Feroniki Adamidis, Franziska Ecker, Jessica Stöger, Eva Katharina Masel

**Affiliations:** 1 Division of Palliative Medicine, Department of Medicine I, Medical University of Vienna, Spitalgasse 23, 1090 Vienna, Austria; 2 Department of Psychoanalysis and Psychotherapy, Medical University of Vienna, Spitalgasse 23, 1090 Vienna, Austria

**Keywords:** Palliative care, Education, Medical humanities, Patient-centered care, Graphic medicine

## Abstract

**Objective:**

The aim of this study was to create a patient-centered comic on palliative care with and for patients admitted to a palliative care unit and to examine their experiences of graphic educational material.

**Methods:**

This study employed a qualitative methodology using semi-structured interviews with advanced cancer patients admitted to the palliative care unit of the Medical University of Vienna. The data were analyzed using thematic analysis with the assistance of the MAXQDA software.

**Results:**

In the first phase of the comic creation 6 interviews were conducted and 15 additional interviews in the second phase, all of which examined patients’ reactions to the comic. The findings revealed three themes: 1) patients’ experiences with and understanding of graphics about palliative care, 2) patients’ perceptions of the possibilities for use of the comic and 3) how the visual material facilitated access to the patients’ shared imagination and interpretations. The medium generated curiosity, participation, and positive reactions. Patients were inclined to be involved in patient-centered educational material that enabled an entry into conversations and connection, giving access to feelings and associations of patients.

**Conclusion:**

The results of the present study offer insights into mostly positive reactions of patients when presented with a comic about palliative care. The study showed how illustrated educational information led to deepened conversation among the interviewer and the patients, offering insights into their experiences and imagination.

## Introduction

For many people palliative care (PC) is an abstract expression. PC is an essential aspect of medical care, yet there are still gaps in its integration into the medical system on a global scale and reluctance among patients to use it. To improve PC utilization, information on PC needs to be more visibly displayed in areas where cancer patients are treated [1].

It was shown that 39.8% of patients would feel anxious if referred to PC [2]. Patients often associate the term PC with hopelessness, resignation and dependency, which can lead to fear and avoidance [3–5]. These findings call for improved patient education about PC.

Medical information provided by physicians is known to be poorly remembered by patients, especially when it comes to topics provoking anxiety [6]. It is therefore essential that further research is conducted on how to best educate people about the benefits and scope of PC. Creative methods to communicate such information can provide an appropriate and accessible education.

Comics as a communication medium are characterized by the simplification of complex content [7]. Graphic medicine is defined by the “intersection between the medium of comics and the discourse of healthcare” [8]. This includes the use of comics in medical education, expression of patients’ and caregivers’ experiences and also to supplement patient care [9, 10]. Studies have repeatedly demonstrated that integrating pictures with text improves comprehension. This particularly aids in the retention of health-related information, especially among individuals with low literacy levels [11]. Moreover, the effectiveness of this medium is heightened when pictures clarify the text, making the content more engaging and enhancing patient attention, understanding, memory retention and adherence [12].

Medical comics in patient education showed significant positive results in a recent German study using medical graphics to illustrate information for cardiac catheterization examinations [13]. Patients who were given a comic with visual information before the procedure showed greater comprehensive understanding and less fear about the procedure. Also, the World Health Organization shares a comic-influenced video to explain PC online [14], and in a recent article, Zimmermann and Mathews used a comic to visualize a metaphor to guide conversations on late vs. early PC [15]. Comics represent great potential to improve patient education about PC. This study is the first to investigate the creation and use of a medical comic among PC patients.

## Patients, material and methods

This study consisted of two interview phases: round A and round B. A qualitative approach was utilized, employing semi-structured interviews with predetermined open-ended questions (Tables 1 and 2). This allowed participants to express themselves freely, emphasizing aspects they deemed important, while still providing a structured framework and ensuring that crucial topics were not overlooked [16].

**Fig. 1:**
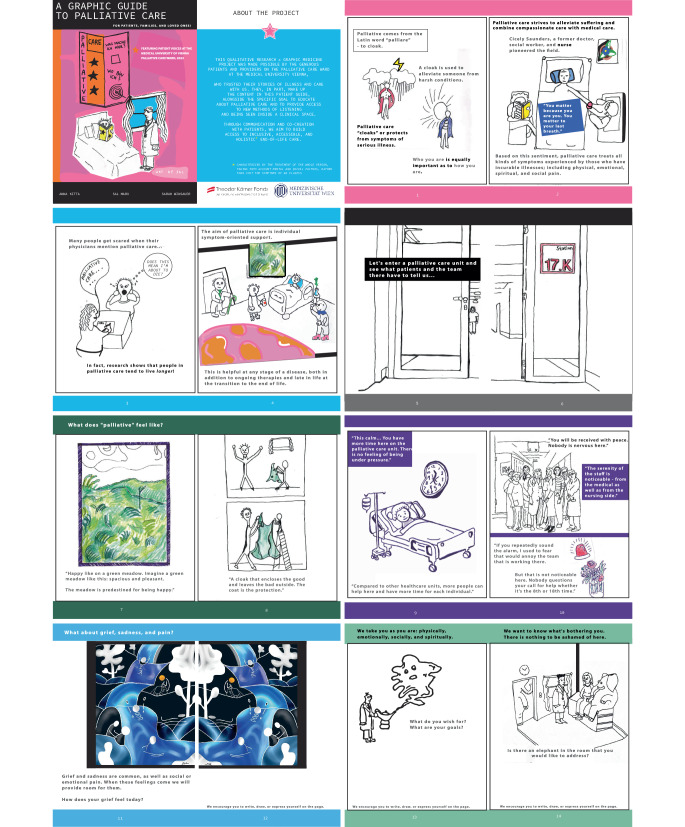
English version of the comic *A* *Graphic Guide to Palliative Care*

**Fig. 2:**
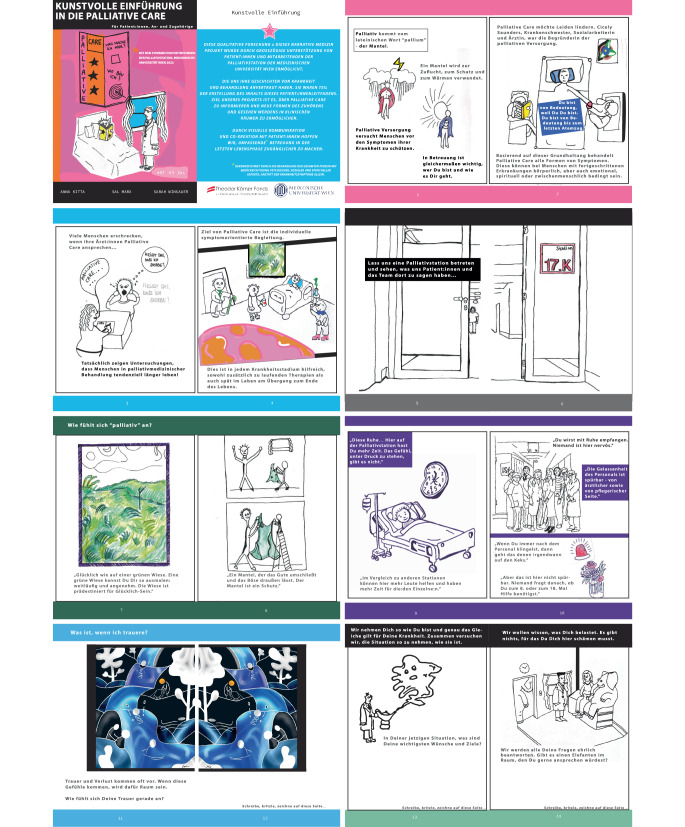
German version of the comic *A* *Graphic Guide to Palliative Care*

In round A, initial interviews were conducted with patients on the PC ward to explore their experiences, perceptions, and ideas about PC. These interviews served to capture the perception of the patients and to incorporate their thoughts and feelings as a basis for the creation of the comic. Once enough information had been gathered, SM, a visual artist and member of the research team, took the focal points identified by the patients and creatively developed the comic “*A Graphic Guide to Palliative Care*” (Figs. 1 and 2). In round B, the comic was given to patients on the PC unit, and they were subsequently interviewed using interview guide B (Table 2). Accompanying the interview, additional observations were noted by the interviewer, including patients’ reactions, emotions, and other forms of non-verbal communication.

### Participants and data collection

The study was conducted at the PC unit of the Medical University of Vienna, a 12-bed hospital-based facility that primarily serves patients with advanced oncological diseases. Inclusion criteria were age 18 years or older, absence of barriers to participation (e.g., cognitive impairment, severe mental illness, physical limitations), absence of language barriers, and ability to provide written informed consent. Patients who did not meet the inclusion criteria or who refused consent were excluded. Interviews were conducted between April and September 2023. To minimize bias, all interviews were conducted by a medical student (SW) who was not a member of the clinical team. Ethical approval was obtained from the ethics committee of the Medical University of Vienna (2034/2022).

### Data analysis

The study adhered to the consolidated criteria for reporting qualitative research (COREQ-32) [17]. Interviews were digitally recorded and transcribed verbatim, conducted in German, and translated into English by the authors.

Round A: data of the initial interview phase were coded, and significant quotes were highlighted; no complete thematic analysis was conducted. Pertinent themes were creatively integrated into the comic. Ideas were discussed by AK, SM, and SW, with the final illustrations executed by SM.

Round B: thematic analysis was conducted with the interview transcripts from round B, which involved patients who had either read or were reading the comic. Thematic analysis is suitable for examining descriptions of experiences [18–21]. The coding process began after the initial interviews were transcribed and continued throughout the analysis phase. The data were analyzed using MAXQDA software (VERBI Software, Berlin, Germany) and two researchers (AK, SW) independently generated a list of codes based on participants’ responses after reviewing the initial interviews and their findings were compared. Consensus was reached through group discussions involving two additional researchers (SM, EKM) to resolve minor discrepancies. The interview process continued until theoretical saturation was reached, indicating that no new codes were emerging from subsequent interviews. To confirm this, an additional interview was conducted and, as no new codes emerged, the interview phase was terminated. Transcripts were not returned to participants for feedback or correction. Themes were identified, reviewed, defined, and named by team consensus.

## Results

### Patient characteristics

During round A six patients were interviewed (Table 3). The conversations lasted 5–62 min (mean 31 min). One participant ended the interview early as he experienced that his language proficiency was not sufficient for the interview. Round B was performed with 15 participants (Table 4) and lasted 12–52 min (mean 31 min).

All six patients of the primary phase of the study lived with advanced cancer and were included consecutively. Demographic characteristics of included patients were as follows: 1 woman, 5 men; median age 67 years (range 51–88 years). During the main phase of the study and data collection 15 interviews were conducted with different patients than those in the first phase. In round B, eight participants were female, seven were male. Patients were again included consecutively, 14 patients lived with advanced cancer and 1 with progressive gastrointestinal disease. The median age range of this group was 69 years (range 58–89 years). Of the 21 interviews 2 were conducted in the presence of the caregivers (P05A, P15B).

These results are presented and analyzed in this article. The participants of the interviews from round A are marked with the letter A (e.g., P01A for the interview with patient 1), whereby interviews from round B are marked with the suffix B (e.g., P01B is a different person than P01A). An overview of all interviews conducted can be found in Tables 3 and 4.

### Co-creation of the comic

The main results of the first interview phase (round A) shed light on topics that patients found important regarding PC. These topics can be summarized as fear, ignorance, difficulties in explaining the term, characteristics of PC as a medical scientific specialty, and PC as something positive and free from pressure.

Four patients reported from their own experience that they felt frightened when they heard about the possibility of being admitted to the PC unit (P01A, P02A, P05A, P06A). For example, patients who recalled the moment when they were confronted with the possibility of being admitted to the PC unit shared in the interviews “Ok, shock (voice breaking). But obvious from the physical condition (cries)” (P02A) and “(t)he first time I heard this; I naturally thought it was an expression for the beginning of the end. So, an accompaniment (…) into death. (…) It scared me at the time because it was a wake-up call, ‘hello, you’re on your way’” (P06A).

However, during the interview, this initial trepidation was no longer prevalent in any of the patients. Instead, they associated PC with positive experiences. Four of the six interviewees found answers to the question of how they would inform their roommates or relatives about PC. However, P01A stated that he found it challenging to explain what it was about. Similarly, P05A found it “difficult to convey the message of ‘you’re on your final path’”, P02A described PC as improving the situation to such an extent that life becomes worth living again and P06A found it to be “a great help”.

Subsequently, patient quotes and the main topics from interview round A were reflected on by the research team and illustrated by SM in the form of the comic booklet *A Graphic Guide to Palliative Care* (Figs. 1 and 2). The content was supplemented with important aspects related to the authors’ clinical experience in PC, such as the origin of the word “palliative” and historical information about the field. For example, information on Cicely Saunders, the pioneer of palliative care and a former doctor, social worker and nurse was added. Additionally, open-ended questions and opportunities for creative expression were included to encourage patient engagement. The authors created both English and German versions.

### Qualitative findings

#### Theme 1: patients’ experiences with and understanding of the graphic material on PC

##### Initial associations with comics and triggered feelings

Nearly all patients reported having read comics before and expressed a positive attitude towards this medium. Many mentioned enjoying comics during their childhood and youth, citing examples such as Mickey Mouse, Fix and Foxi, or Asterix and Obelix.

The comic material elicited various emotional reactions. Some patients quickly flipped through it and put it away (P13B), which might suggest a lack of interest or defense against emotions that the content triggered. Others expressed being deeply moved by certain passages (P01B). Some disagreed with parts of the content (P03B), while others either cried (P04B) or laughed (P09B). Many patients later verbalized their emotions. Patient P13B nodded to the question whether it made her sad, and confirmed by stating, “In a way, yes... It’s not funny” and P11B also found the booklet “depressing”. In contrast, P07B smiled while reading and P04B had tears running down her face at one point, but ultimately concluded that it made her “feel lighthearted”: “cheering up, yes, ultimately it was mostly funny”.

##### Positive and critical evaluation

Most of the patients expressed appreciation for the project, asked to keep the comic, or requested additional copies for their loved ones. Also, P01B stated “this is the best thing I’ve heard in the last 20 years. You’re definitely hitting the mark with this activity”. It is noteworthy that readers frequently had a positive reaction when they recognized themselves or their experiences in the brochure. Reactions included: “yes, yes that’s true” (P02B, regarding page 9 and more time on a PCU); or “that’s also difficult for me” (P03B, regarding page 11/12 on grief and loss). P01B found the image of the coat appropriate and noted feeling protected in the PC unit. P10B exclaimed: “this is so close to reality, (…) with how time passes, with the clock there”. Above all, it was mentioned repeatedly that the booklet had a very informative character and that the content was of importance (e.g., P04B, P07B).

Patients expressed the benefits of visual communication in the booklet. For one participant, the comic made information “concise and crisp” (P07B). Others reiterated that the combination of text and images has also led to a better understanding (e.g., P10B). According to P11B, the images provided greater clarity as he felt they were more concrete than language. P13B confirmed that the images conveyed more than text alone. P09B agreed to prefer pictures over reading lengthy text, saying simply, “I am a picture person.” The use of images and metaphors had recently helped this patient understand her medical condition. She shared that a doctor had used an analogy to explain the consequences of her tumor in her abdomen like a blocked street inside her: “and for me it’s much easier to understand than if he somehow explained it to me medically”. P11B summarized the potential for art to serve as a means of self-expression and articulation, regardless of the medium.

The patients also provided strong opinions on what they would change in the comic. P11B pointed out that an important aspect of patient care was missing: “The most important message of this comic should be that the focus is not on the illness, but on the person, the patient”. P03B found it difficult that end of life was mentioned and expressed that the authors should be careful with such wording. He stated, “If someone doesn’t know that now or doesn’t know it at all, then they might think they’re going to die here”.

Another piece of critical feedback came from a patient and former internist who was skeptical about the effectiveness of graphic material. He stated, “I believe that a conversation is much more important. I am convinced of this and would never choose a comic to explain things to patients. Instead, I would sit down with them and try to make them understand to the best of my knowledge and ability” (P12B). He added that comics can lack direct interaction, which was crucial for understanding patients’ reactions. Patient 19B agreed that conversation is preferable to reading a comic alone. The participant expressed difficulty in her reading literacy and emphasized the importance of verbal communication.

The feedback included critical remarks about specific images (P14B), the use of certain colors (P01B, P04B, P06B, P15B), and the use of gender-inclusive language within the German version (P14B, P03B). Additional feedback indicated that humor was important (P04B), while also noting that the comic lacked humor and was too serious (P05B).

##### Difficulties to understand

During the interviews it became evident that certain patients had difficulty comprehending the intended meaning of the booklet. This was due to certain ambiguous visual representations, such as interpreting the spiral on the title page with a washing machine (P02B) or across sections that lacked clarity in reading the text. For example, P08B asked: “Can you tell me what ‘palliative’ is? I don’t know the word, I’ll tell you now. And what does it mean?” Additionally, the metaphor of the elephant in the room (page 14), which was intended to stand for a problem that is not openly expressed, at times caused confusion among participants (e.g., P11B), as the English expression “an elephant in the room” does not exist in German. However, the image was interpreted in many different ways (e.g., death, transitioning to another world, family).

#### Theme 2: patients’ perceptions of the possibilities for use of the comic

The patients provided their input on potential uses for the visual information material. Additionally, the conversations themselves revealed additional potential areas of application.

##### Possible target groups and early education/information

During the interviews the topic of the target audience was raised. P07B expressed uncertainty about which age group was the intended group to be addressed with the material. She proposed: “I believe that the generation who should be addressed have a sick grandmother or who have a cancer patient in the family. So, I think young people don’t even know what PC means. I’ve also seen it with my sister-in-law, I just said PC and she was terrified. I said, ‘Why?’ ‘Well, that’s where people go to die’. I say, ‘Well, people don’t go there to die’.” Additionally, other respondents suggested children as a potential target audience. P14B viewed the comic as an attempt to explain PC in an “understandable way that children can also understand.”

Patients reported being uninformed about certain aspects of the material and found it informative (e.g., P02B). P07B believed it was important for the general population to read it because they did not deal with death enough. Others saw the benefits primarily for those affected personally. P02B found it “incredibly helpful” and “relieving” as it got to the heart of the matter.

##### Reducing anxiety and starting a conversation

Interviewees saw the use of visuals as being effective in reducing anxiety related to PC. P01B explained that she felt she recognized herself in her fear through the comic, which helped to reduce it. P02B believed that it could benefit others who experience anxiety about entering the PC unit. P04B expressed that the comic could have been helpful earlier in her illness: “It may have avoided the anxiety to some extent or addressed it earlier because at one point you have to deal with it.”

The comic was successful at initiating conversations about PC. The interviews showed how the visual material facilitated interactions and that the participants themselves began to ask the interviewer questions. For example, one patient was very pleased to read that a study had shown that patients treated with PC lived longer, and when he encountered Cicely Saunders in the text, he immediately asked, “How old did she get?” (P02B).

A key finding was that the material proved useful for talking about topics for which words are sometimes lacking. Specifically, multiple participants found the pages on grief to be particularly helpful. P01B admitted that while she might not have brought up the topic on her own, she believed the page on grief was important and had personal experience with it: “I also think grief is quite valid.” Additionally, participants emphasized the importance of discussing death and dying (P06B) rather than the tendency to avoid the topic altogether (P04B).

It was also noteworthy that nearly all participants emphasized the potential use of comics to discuss what PC means with their loved ones. Some requested additional printed comics to share with specific individuals, such as their partner, sister or son (P01B, P08B). P04B summarized the topic:

“It’s not that you’re burdened with it yourself, it’s more the relatives who are burdened. (…) I think that PC should be primarily focused on the relatives of the patient. This is because, (…) when one is ill, it is inevitable that one will eventually die. (…) I have found that I am better able to cope with this knowledge than my relatives.”

Ultimately, participants suggested that the comic could provide needed information for family and friends and create an introduction to difficult conversations.

#### Theme 3: how the visual material facilitated access to the patients’ shared imagination and interpretations

The process of reviewing the information material together with the patients yielded another unexpected insight. The visual material facilitated access to personal aspects of the participants as it enabled the observation of their own power of imagination.

It was enlightening to read the material directly to patients or with participants, who could then highlight certain pages or point to images. As patient and interviewer sat together, the joint viewing enabled the observation that participants occasionally perceived the images in a manner that differed from the intended meaning. While sharing their associations of looking at the drawings, they provided insight into the cognitive processes of being affected, revealing their inner worlds, their needs, all of which facilitated the discussion of previously unspoken or repressed topics, including hopes.

During the interviews, numerous associations with the drawings in the comic were offered. The visuals were enriched with various personal associations that held meaning for specific patients. For instance, P04B interpreted the small window (page 8) as her perspective on her own illness.

**P04B**: “The small window is so far away for me. That’s the way it is, but at some point, you have to deal with it”.

**I**: “What is the small window for you?”

**P04B**: “That little window, that little coat, it’s always so far away for me, in terms of the image now. But it’s this big coat that’s put over it again. And it has to be put over it one day, yes.”

**I**: “What does it have to be put over?”

**P04B**: “Over the illness. So, you have to deal with it at some point. (…) That’s how a protective layer is created over it.”

**I**: “And what’s further away from the small window?”

**P04B**: “Well, the protection, the acceptance that it’s there now, the acceptance that the illness is there.”

This example and similar statements indicated a profound, emotional, spiritual, and imaginative engagement with the educational material. Speaking on the experience of imagination, P11B stated:

“So, if I have a reader, for example, who has a lot of imagination (…), then I don’t need a lot of images to take them on a journey. I mean, of course it can always happen that they (…) then end up somewhere completely different to where I, as the author, think they should be. Of course, it could be that he’s taken such a wrong turn in his imagination that I didn’t even think it was a possibility.”

He thus indicated that the given texts and images met the patients’ individual imaginations and that authors could not know nor plan where the material would take the reader mentally.

## Discussion

This study found that visual material deepens education about PC and can facilitate difficult conversations. As King stated, comics can convey specific knowledge and individual experiences that are essential for certain health topics [22]. The findings suggest that using images may be more effective in situations where words are insufficient. Rakower et al. illuminated this by demonstrating that the medium is particularly useful for personal topics that people are reluctant to discuss in public or at all [23]. Although their study focused on communicable diseases, it is also applicable for topics such as death and dying, experiencing oneself as a burden, or experiencing debilitating symptoms associated with shame.

As these previous studies suggested, this current paper also finds that creating comics can help patients better understand medical content. Furthermore, comics might allow healthcare professionals to foster conversations with patients, their friends, and families, and to understand their knowledge, feelings, or needs better. The comic was not intended to replace informative discussions with an oncologist or palliative care specialist but to enable or enhance them. Further research is necessary to understand the differences in experiences or willingness to engage with graphic material amongst age, racial, and class demographics.

The interviewees closely engaging with the comic material, both their critical remarks and their affirming experiences, gave insights into how graphic content should be prepared in detail and strive to be universally recognizable. Simultaneously, this research also highlights the limits of attempting a one-size-fits-all graphic representation. With any range of people, cultures, bodily and mental experiences and illnesses, it is harmful to assume there is only one way to represent all stories accurately and ethically in a single graphic. As such, this project strived to include graphic sections with intentional blank spaces, for the person reading to engage with and to communicate on the page what feels right to them. Ultimately, these findings indicate the importance of balancing content across the spectrum of emotions, including the positive effects of PC and experiences of grief, sadness, anxiety or anger.

The booklet of this study uses text and images, and through the different languages of the research team, we experienced how dividing language barriers can be. Similarly, one patient terminated the interview after a brief period, citing the inability to engage in a meaningful discussion due to his perceived lack of linguistic proficiency in German. Despite his familiarity with German, this was a crucial barrier for him to navigate. Comics can counter the commonly seen language barriers in medical spaces, through universally understandable visuals.

It was an unexpected finding that the respondents’ imagination was stimulated through the visual material. The participants often independently linked the comic content with their individual feelings and ideas, thereby creating new pictures and connections. These images contained a part of the personal world of thought of those affected. Statements that revealed these imaginations were therefore a valuable tool for the researchers to gain insights into the experience of the comic and might be a valuable insight for clinicians when using the material in clinical contexts.

### Strengths and limitations of the study

This study is one of the first to use an approach to create educational comic material together with patients who are in a PC setting. This makes it especially worthy as it contains the insights of those who receive PC. However, the scale of the six participants who initially shared their perspectives on PC is small, and it is a predominantly male perspective. This resulted from the consecutive inclusion design of the study. Additionally, persons with a less positive perception of PC might have been less willing to participate in the interview round A of the study. Furthermore, certain observations in this study are unique to Austrian culture and may not be representative in all hospital settings on a global scale. The critical feedback of the patients could be valuable for future studies and the development of additional graphic material.

## Conclusion

Using visual material to inform about PC may present a variety of different reactions. The results of this study show that the medium itself sparked curiosity, engagement and positive reactions, highlighting that patients are inclined to be involved and share additional ideas for improvement in patient-centered educational programming, which could be used for the generation of further graphic material on PC. Most importantly, this study shows that graphic educational booklets work as a successful entry into important conversations that might be difficult to initiate. Ultimately, we have seen that comics present an opportunity for further connection, a platform to access feelings and associations, all of which foster the imaginative world of patients and caregivers.

## Appendix

### Interview guides

**Table 1:** Interview guide A

Today’s interview is about what you think is important to know about palliative care and what information you would have liked to have before being admitted to the palliative care unit
Where were you treated before you were admitted to the palliative care unit?
Tell me about the time when you were being considered for admission to the palliative care unit.
What information about palliative care were you given?
What do you think is important to know about palliative care?
Can you tell me about moments when you learned important aspects?
What do you wish you had known before you were admitted to the palliative care unit?
If you had to explain to another patient what a palliative care unit is, how would you do it?
Has anything about palliative care frightened you?
Were there prejudices against palliative care?
What made you trust palliative care?
What surprised you during your stay at 17K?^a^
Is there anything else you would like to add?
Is there anything I can do for you right now?

^a^17K is the name of the PCU of the Medical University of Vienna, where the interviews took place.

**Table 2:** Interview guide B

In this interview I would like to show you a comic and ask you for your opinion.
How do you experience the comic?
What does this comic mean to you?
Can you describe the emotions this comic makes you feel?
Did you learn something new from the comic that you didn’t know?
How do you feel about palliative care in general?
Optional: Earlier you described the feelings [insert feeling] when reading the comic. Does this feeling influence your general thoughts about palliative care?
Do you think this comic could reduce fears and prejudices about palliative care?
What do you think about comics in general?
What benefits do you see in the comic? What risks do you see if the comic was used in a clinical setting?
What do you like about it? What do you like less?
Have there been situations in your illness where the comic would have helped you?
What effect do you think it would have on other palliative care patients to see this comic before admission?
Is there anything else you would like to add?
Is there anything I can do for you right now?

**Table 3:** Profile of the study participants A

Participants	Age (years)	Gender	Oncological disease	Months since first diagnosis
P01A	88	male	Colon cancer	8
P02A	51	male	Sarcoma	5
P03A	82	male	Lung cancer	5
P04A	50	male	Rectal cancer	33
P05A	72	male	Lung cancer	3
P06A	60	female	Colon cancer	60

**Table 4:** Profile of the study participants B

Participants	Age (years)	Gender	Oncological disease	Months since first diagnosis
P01B	77	female	Liver cancer	44
P02B	58	male	Lung cancer	9
P03B	76	male	Glioblastoma	7
P04B	56	female	Lung cancer	21
P05B	78	male	Urothelial cancer	24
P06B	82	female	Stomach cancer	5
P07B	52	female	Lung cancer	21
P08B	82	female	Pancreatic cancer	3
P09B	62	female	Small intestine cancer	10
P10B	69	female	Pancreatic cancer	2
P11B	57	male	Urothelial cancer	10
P12B	89	male	Lung cancer	114
P13B	76	female	Stomach cancer	39
P14B	43	male	Thyroid cancer	29
P15B	75	male	Liver cirrhosis	8
